# A remotely accessible plant-based culinary intervention for Latina/o/x adults at risk for diabetes: lessons learned

**DOI:** 10.3389/fnut.2024.1298755

**Published:** 2024-02-13

**Authors:** Linda M. Koh, Favorite Iradukunda, Airín D. Martínez, Keila C. Caetano Schulz, Irene Bielitz, Rae K. Walker

**Affiliations:** ^1^Stanford Prevention Research Center, Department of Medicine, School of Medicine, Stanford University, Palo Alto, CA, United States; ^2^Elaine Marieb College of Nursing, University of Massachusetts Amherst, Amherst, MA, United States; ^3^School of Public Health & Health Sciences, University of Massachusetts Amherst, Amherst, MA, United States; ^4^Independent Researcher, Loma Linda, CA, United States

**Keywords:** plant-based, diabetes, Hispanic/Latino, health equity, community-engaged, nursing, culinary education, remote interventions

## Abstract

**Introduction:**

Little research has examined how community-engaged and -participatory dietary interventions adapted to remotely-accessible settings during the COVID-19 pandemic.

**Objectives:**

To identify lessons learned in design, implementation, and evaluation of a remotely-accessible, community-based, nurse-led approach of a culturally-tailored whole food plant-based culinary intervention for Latina/o/x adults to reduce type 2 diabetes risk, delivered during a pandemic.

**Methods:**

A mixed methods quasi-experimental design consisting of a pre-post evaluation comprised of questionnaires, culinary classes, biometrics, and focus groups.

**Lessons learned:**

Community partnerships are essential for successful recruitment/retention. To optimally deliver a remotely-accessible intervention, community leadership and study volunteers should be included in every decision (e.g., timeframes, goals). Recommendations include managing recruitment and supply chain disruption of intervention supplies.

**Conclusion:**

Future research should focus on increasing accessibility and engagement in minoritized and/or underserved communities, supply chain including quality assurance and delivery of services/goods, study design for sustainable, remotely-accessible interventions, and health promotion.

## 1 Introduction

This study focused on Latina/o/x adults living in the Inland Empire (IE), composed of Riverside and San Bernardino Counties, in Southern California, where 49% of the IE population self-identifies as Latina/o/x with the majority being of Mexican origin (1). A recent report estimated that 46% of California’s population has prediabetes with Latina/o/x adults having a 50% chance of developing diabetes during their lifetime (2, 3). In the United States, Hispanic/Latino individuals are 1.4 times more likely to die from diabetes than non-Hispanic whites due to social determinants of health and systemic oppression due to allocation of power and resources (4–9). Every year more than $400 billion are spent on diabetes medication and symptom management (10). While physicians and dieticians often provide guidance on dietary choices and nutrition (11, 12), nurses also receive education and training on providing resources for making informed healthcare decisions (13), including those related to nutrition and diabetes.

Originally intended as a face-to-face culinary intervention, due to the pandemic and need for physical distancing, the redesign of this study shifted to a remotely-accessible format. The redesign included meetings and multiple conversations with community members and leaders, advisors, as well as pretesting study volunteers. These discussions focused on the delivery format of research activities and culminated in implementation of a nurse-led, culturally-tailored, WFPB intervention in a remotely-accessible environment for Latina/o/x adults at risk for developing T2DM. Selection of the WFPB intervention was based on community feedback and previous research showing that a WFPB diet is economical and sustainable for individuals and the planet (e.g., potential to prevent or reverse diabetes, lower blood pressure, promote insulin sensitivity) (14–17).

To maintain accountability to the study volunteers, communities, and other stakeholders involved or potentially impacted, a community advisory board (CAB) group consisting of community members and leaders was consulted during the design, implementation, and data analysis stages. While several of the CAB members worked in healthcare centers, schools, and other local organizations and businesses, some of the individuals were working from home or unemployed. Initially, the CAB members met individually with the primary investigator (PI) on a weekly basis or in groups of up to three individuals. Once the study commenced, meetings occurred every 2 weeks. To enhance trustworthiness, address power in the research process, increase accountability and integrity, community partners from the IE were also engaged as reflexivity partners during the data analysis phase for member-checking.

### 1.1 Aim

The purpose of this paper was to identify lessons learned in design, implementation, and evaluation of a remotely-accessible, community-based, nurse-led approach of a culturally-tailored whole food plant-based (WFPB) culinary intervention for Latina/o/x adults to reduce type 2 diabetes risk (T2DM), delivered during the COVID-19 pandemic, over a six-week period (January–February 2022).

## 2 Objectives

The specific aims of the study included (1) to explore the feasibility, safety, and accessibility of a remotely-accessible WFPB culinary and dietary intervention and (2) to assess the WFPB intervention on knowledge and self-efficacy for making healthy WFPB food choices with assessment at baseline and post-intervention. Guided by the PRECEDE-PROCEED Model (18), this remotely-accessible intervention included learning about WFPB food preparation and nutrition while collecting and self-monitoring biometric data.

## 3 Methods

After receiving approval from the University of Massachusetts Amherst Institutional Review Board, the quasi-experimental study commenced in January 2022. The target population was individuals that self-identify as Latina/o/x adults from the community, ages 18–65 years old, who were determined to be at risk for diabetes or prediabetic as determined by the American Diabetes Association Risk Tool (ADART) (19). Individuals were recruited by word-of-mouth and a flyer. During the design of the study and questionnaires, the researchers, CAB members, and pretesting group discussed the term, “Latinx.” This term appeared during the mid-2000s from Latinx lesbian, gay, bisexual, transgender, and queer (LGBTQ) communities in the U.S. as a gender-neutral term (20). Supporters of the term state that it is inclusive of all genders (20). Some critics of the term state that “Latinx” is difficult to pronounce, a colonialist residue, does not follow traditional grammar, and only used by 3% of the population (21). However, it is important to also remember that gender neutrality does not necessarily equal gender inclusivity and a catch-all term may cause unintended marginalization (22). Therefore, in this study, the researchers, CAB members, and pretesting group agreed that the term “Latina/o/x” would be used, with the exception of the manuscript introduction where “Hispanic/Latino” was also used. In addition, Table 1 includes a footnote stating that study volunteers were provided with additional options, on questionnaires, such as non-binary or not listed, including write-in and prefer not to reply, but none of these were selected by respondents. After providing informed consent, study volunteers (Table 1) self-selected to join one of two groups. Study volunteers in the WFPB intervention group (*n* = 15) verbalized an interest in learning more about WFPB nutrition, T2DM prevention, and cooking WFPB recipes. They participated in culturally-tailored WFPB culinary classes, through videoconferencing, adapted and based on the “Culinary Medicine Curriculum” from the American College of Lifestyle Medicine, originally designed by physician and chef, Dr. Michelle Hauser (23). Additional WFPB recipes were adapted from “Decolonize Your Diet,” a plant-based Mexican-American cookbook (24) The second group (*n* = 6) also met through videoconferencing, as a comparison group (ADA), and received standard care using the “CDC Prevent T2” curriculum and American Diabetes Association recommendations for diet and meal planning (25, 26).

**Table 1:** Study volunteers’ characteristics (*n* = 21).

Characteristic	Description	Whole food plant-based (WFPB; *n* = 15)	Standard care (ADA; *n* = 6)
Age*	18–40	5 (33%)	1 (17%)
	41–65	10 (67%)	5 (83%)
Gender/Gender Identity**	Women	11 (73%)	4 (67%)
	Men	4 (27%)	2 (33%)
Relationship Status*	Never Married	2 (13%)	1 (16.7%)
	Married	11 (73%)	4 (66.7%)
	Domestic Partnership	1 (7%)	–
	Divorced	1 (7%)	1 (16.7%)
Income Level*	$0–49,000/year	5 (33%)	2 (33.3%)
	Above $50,000/year	7 (47%)	2 (33.3%)
	Refused/Unknown	3 (20%)	2 (33.3%)
Level of Education	Less than High School (HS) degree	–	1 (16.7%)
	HS/Gen Ed Development (GED)	4 (26.6%)	2 (33.3%)
	Some College	4 (26.6%)	–
	4-Years of College or Higher	7 (47%)	3 (50%)
Family History of Diabetes	Yes	14 (93%)	4 (67%)
	No	1 (7%)	2 (33%)
Personal History of Hypertension	Yes	5 (33%)	2 (33%)
	No	10 (67%)	4 (67%)

^*^Study volunteers were provided with options for age, as well as income ranging from $0–100,000+/year. Both categories were dichotomized to protect study volunteers, due to decreased variability in responses. Also, the majority of study volunteers had health insurance, but due to decreased variability in responses, this information was not included in the table. **Study volunteers were provided with additional options such as non-binary or not listed: write-in, prefer not to reply, but none of these were selected by respondents.

Previous studies have used the PRECEDE-PROCEED Model to evaluate the effectiveness of community-based health programs, as well as design, implementation, and evaluation of interventions (18, 27, 28). During the PROCEED phases, identification of desired outcomes and program implementation were evaluated based on reflection questions such as, “Are you doing the things you planned to do?” “Is the intervention having the desired impact on the target population?”

Using the Model as a guide, each approach (e.g., WFPB, nurse-led, culturally-tailored) was reviewed and evaluated. This analysis was designed, in part, to help answer questions in the context of collaborating with the Latina/o/x community disproportionately impacted both by the pandemic and by systems of oppression and marginalization that place them at higher risk for negative health sequelae such as T2DM.

## 4 Lessons learned: results and discussion

The overall study was well-received with 71.4% of study volunteers reporting that if given the chance, they would participate in the program again. Study volunteers in both the intervention and comparison groups verbalized that they shared the study materials (e.g., recipes, culinary and T2DM prevention resources) with their family and friends. Lessons learned are listed based on the approaches.

### 4.1 Community-based approach

Prior to design and implementation of the intervention, a community assessment, as well as dialog with community leaders and members took place. Community leaders were defined as individuals that had leadership roles or strong relationships with the Latina/o/x communities (e.g., community organizers, healthcare professionals, educators), while also self-identifying as Latina/o and working and/or residing in the IE. Community members were individuals that resided in the area, while also expressing an interest in improving the overall health of the community. These individuals made recommendations (e.g., potential need for a translator, time of year to implement the study) to the PI for inclusion. Recommendations were then integrated into the overall design. This provided opportunities to build trust, as well as create an inclusive environment for the community to voice concerns, provide feedback, and be involved in the overall process and design.

The PI’s previous work in the IE allowed for long established relationships with community partners that were key for recruitment. Community-and faith-based networks were also utilized for recruitment based on feedback from community leaders and members and previous research (29–35). Although the partnership between the PI, university, and the community had a duration for the length of the study, individuals from the community and the university both expressed interest in potential future collaborations.

### 4.2 Nurse-led approach

Previous researchers reported that Latina/o/x individuals preferred participating in research activities led by healthcare providers (36). Therefore, the PI and the focus group moderator were masters-level registered nurses, multicultural, multilingual, and had additional training and/or certifications in WFPB nutrition and diabetes education. This nurse-led approach provided opportunities for community members to build connections with nurses from the IE with expertise in WFPB nutrition, T2DM, and health promotion.

### 4.3 Culturally-tailored approach

Cultural-tailoring occurs when the structure of a program or practice is reviewed and adapted to fit the needs and preferences of a particular cultural group or community (37, 38). Cultural-tailoring may range from the use of bilingual and bicultural staff, social and community support, to curriculum development that embeds values, practices, and traditions (39). In this study, the curriculum was adapted to include discussion of cultural traditions surrounding identity and food, language, group problem solving, discussions regarding beliefs about family, health and illness (e.g., healthy weight, natural remedies, seeking professional advice), and recipes. Although culturally-tailoring can be beneficial, it is important to acknowledge that people are unique individuals, regardless of group-identification. While WFPB nutritional recommendations are often presented in contrast to a standard American diet, this study included cultural-tailoring of the WFPB culinary curriculum. This resulted in a strengthened curriculum that provided additional opportunities (e.g., using familiar recipes, discussion topics, conducive to beliefs and practices) (40) to reach the Latina/o/x communities. One WFPB intervention study volunteer, that attended more than half of the classes stated, “I think it definitely needs to be tailored to culture, because I think that’s the best way to increase the chances that the person is going to be ‘compliant’” while also stressing the importance of assessing community needs. Study volunteers also verbalized the importance of family and social support, and the impact that support has on lifestyle changes. This feedback reinforced the group format study design that provided a safe space for study volunteers to meet remotely during the COVID-19 pandemic. One study volunteer expressed that the weekly sessions helped them to realize when thinking about health and nutrition, “it is okay to be different from the others” (e.g., lifestyle changes) and five study volunteers shared experiences on making compromises and negotiating with their family members’ choice of restaurants, grocery shopping, and meal planning to follow a WFPB diet. While some study volunteers expressed having support systems in place for lifestyle changes, other study volunteers expressed that even after verbalizing intentions to family and friends, they did not always feel supported.

The researchers emphasized the importance of culture (e.g., customs, family, community, ethnicity and ethnic foodways), as it has the potential to impact all aspects of the study, including recruitment and retention, social support for study volunteers, and building trust.

### 4.4 Remotely-accessible setting

Study volunteers reported that the remotely-accessible setting was feasible, but still posed challenges. Challenges included needing reliable internet access and finding a private workspace that allowed access to both the device screen and cooking area. Some individuals switched from using a desktop computer to a mobile device, but mentioned not always having the ability to view the small screen from a distance and running out of battery.

Although study volunteers selected their preference for class times, it may be beneficial to provide an asynchronous option for future studies. However, some study volunteers reported preferring the weekly synchronous format because it helped with “accountability” and “staying on track.” They also reported that the format enabled participation during the COVID-19 pandemic, so they “felt safe,” with one individual stating, “From my perspective, I do not think I would have participated if it wasn’t online.” The synchronous format also provided the benefit of immediately observing and implementing culinary skills within study volunteers’ kitchens while using their own cooking utensils and supplies.

Some study volunteers requested copies of the slides and recordings to review due to being hard of hearing and stated that they preferred to use the chat feature in Zoom, rather than speaking. Study volunteers often turned off their cameras and microphones during recorded sessions. For this reason, it was decided not to record at all times, but rather during selected sessions or portions. Previous research regarding technology access and use among low-income immigrant Latino parents revealed that individuals frequently used text messaging (40), which might partially explain the preference for the chat feature.

### 4.5 Whole food plant-based approach

The PI noted that community members would sometimes receive food assistance, including fresh fruits and vegetables, which they would then request to trade for processed food. When asked, individuals often reported an interest in WFPB nutrition and/or increasing fruit and vegetable intake, but stated were often unsure of preparation of the fresh fruits and vegetables, “so they usually just sit in the back of the refrigerator.” Knowledge from these conversations was included in the design.

During the study, study volunteers discussed ways to increase their weekly fruit and vegetable consumption, discovered new foods, reported enjoying “cooking on Thursday evening means that I have food for the weekend,” attempted preparing and eating additional WFPB meals during the week, and tried “to learn how to plan ahead.” By providing study volunteers with resources on batch cooking and cooking on a budget, individuals gained knowledge on preparing one versatile item, for example, quinoa, and then using the quinoa to create five different main dishes.

In addition, study volunteers shared multiple recipes with individuals outside the study. Recipes were described as meeting the goal of food preparation: “healthy and delicious.” Because mealtimes are often communal, one lesson learned was that the recipes must be delectable to those that the person prepares food for, not just the individual.

### 4.6 Focus groups

Two voluntary remote focus groups were included as part of the WFPB intervention assessment. The 60-min focus groups provided an opportunity for the study volunteers to discuss their lived and study experiences, including challenges. The ADA standard care group had discussion times within their weekly classes. The first focus group (*n* = 5) met in week four and the second one (*n* = 2) met a few days after the intervention concluded in week six. Using a semi-structured interview guide, a registered nurse trained as a diabetes educator and a student nurse research assistant moderated the focus groups.

Two research team members read the transcripts and independently identified common themes using selected comments as examples for each theme. One team member coded the transcripts, line-by-line, with constant comparison with a member of the research team, using descriptive thematic analysis and *in vivo* coding in NVivo, version 12. By using *in vivo* coding, study volunteers’ actual spoken words and phrases were used as codes. Codes were grouped and clustered into categories. From these categories, themes and concepts were identified as those repeatedly or consistently discussed in relation to the study objectives and indicated in the codebook. Memoing, constant comparison, and close member check-in resulted in the finalizing of the categories and main themes.

Topics covered perceptions of health/illness, current dietary habits, experiences of Latina/o/x individuals, intervention and accessibility, and personal vision and goals related to their health. The focus groups were conducted in English with a trilingual (English, Spanish, and Portuguese) focus group moderator, not the PI. Future focus group study volunteers may benefit from a printed list of the discussion questions beforehand, as well as incorporating elements of photovoice to increase engagement. Study volunteers expressed enjoying sharing photographs of prepared food as a way of promoting conversation and receiving recognition for the creativity they used to engage in the intervention.

### 4.7 Budget

This research was conducted as part of a dissertation research project with study volunteers’ biometric equipment funded by Beta Zeta at-Large Chapter of Sigma Theta Tau International Honor Society of Nursing. The disruption of the supply chain during the implementation of the study impacted the price of the study volunteers’ equipment as prices increased from the time the grant was submitted and obtained. Study volunteers were responsible for costs associated with purchasing their own food items and cooking supplies for the culinary classes. For future studies, it is recommended to proactively create a detailed budget that takes into consideration food cost inflation. Food items that study volunteers and the PI purchased for the culinary sessions (~$25-30/week, depending on items already available at home and where the items were purchased), as well as the cost of equipment and time spent shopping for items also increased. For future studies, it might be more convenient if food items and/or use of a home-based delivery service were provided to reduce participant burden.

### 4.8 Equipment

All study volunteers received glucometer kits, automatic blood pressure cuffs, bathroom scales, and tape measures through the mail. The PI conducted videoconferencing training to teach and/or review use of the equipment with study volunteers prior to biometric data collection. Several study volunteers reported that they had “never paid attention to the numbers at the doctor’s office.” During the WFPB culinary classes and also the ADA standard care sessions, study volunteers were provided with resources and training on parameters for blood pressure and fasting blood glucose, including when to follow up with a healthcare provider.

In some cases, even when using expedited one-or two-day shipping, study equipment was delayed. The U.S. Postal Service reported delays due to a worker shortage and employees in quarantine due to the pandemic. However, there have been previous reports of longstanding issues (e.g., financial and operational), as well as infrastructure underinvestment (41). Based on this information, future remotely-accessible studies should include options to accommodate a variety of study volunteer requests (e.g., asynchronous, additional ways of obtaining biometrics) and/or supply chain disruptions.

While 71.4% of study volunteers completed return demonstrations on equipment use without challenges, 28.6% of individuals reported challenges with the lancing devices (e.g., setup, fear of needles). Post-intervention, 28.6% of study volunteers did not want to use the glucometer, specifically the lancet devices, and did not submit all of their final biometric data. When asked about the lancet devices, one study volunteer stated, “I do not want to poke myself again. I do not know how people with diabetes do this every day.” Previous studies have shown that when providing individuals with training involving needles, it may be beneficial, as was conducted in this study, to provide training at a time that is separate from other activities, using the smallest gauge available, while raising awareness of prevention and treatment options, and discussing any potential barriers (42, 43). Community partners suggested in the future, it might be beneficial to offer the option of a serum lab draw or allowing family members to assist with biometric data collection.

### 4.9 Strength and limitations

The strengths of this study included assessing and evaluating the needs of Latina/o/x adults in the IE with community leaders and members prior to the design. At the time design, consultants with expertise in nursing, community-based participatory approaches, interventions including Latina/o/x individuals, and T2DM provided their invaluable feedback. In addition, prior to the six-week study, all questionnaires were reviewed and completed by the pretesting group. At the close of the study, interested study volunteers were also able to review the findings and results prior to submission for publication. Although the results are potentially transferrable to other contexts and/or settings, this study was specifically designed for Latina/o/x adults residing in the IE.

Limitations of this study include self-reported data, missing data, and time constraints. In addition, community partners and members were not involved in shaping the research instruments. While multiple attempts were made to recover missing data, results may also reflect systematic biases in terms of who was able to complete the study. Based on feedback and patterns in the data collected, there is the potential to expand this study in the future. While qualitative data and use of questionnaires are self-reported, multiple strategies including an audit trail and member-checking activities were used to increase trustworthiness and rigor (44, 45).

## 5 Conclusion

Through this study, the researchers identified lessons learned and found ways to improve implementation of future WFPB remote interventions for Latina/o/x populations. Findings from this research indicate that a remotely-accessible, nurse-led, culturally-tailored WFPB culinary intervention designed to reduce T2DM risk and increase self-efficacy in Latina/o/x individuals is feasible, safe, and enhanced accessibility. Knowledge generated revealed that this type of intervention may not only increase awareness and potentially promote health, but also has potential to increase fruit and vegetable intake among Latina/o/x adults, as long as “healthy and delicious” recipes are provided.

Nurse-led interventions can provide opportunities to explore and gain a better understanding of individual’s beliefs about dietary and lifestyle practices leading to improving future design of nutrition studies. Through identification of risk factors and education, Latina/o/x adults can access additional options and resources for improving their overall health outcomes and making informed healthcare decisions.

## Data availability statement

The datasets for this article are not publicly available due to concerns regarding participant anonymity. Requests regarding datasets should be directed to the corresponding author. The full dissertation with data to support this study will be available through Scholarworks@UMass from May 13, 2027 (see Acknowledgements).

## Ethics statement

The studies involving humans were approved by University of Massachusetts Amherst Institutional Review Board. The studies were conducted in accordance with the local legislation and institutional requirements. The participants provided their written informed consent to participate in this study.

## Author contributions

LK: Conceptualization, Data curation, Formal analysis, Funding acquisition, Investigation, Methodology, Project administration, Resources, Software, Supervision, Writing – original draft, Writing – review & editing. FI: Conceptualization, Methodology, Resources, Writing – review & editing. AM: Conceptualization, Formal analysis, Methodology, Resources, Validation, Writing – review & editing. KC: Conceptualization, Resources, Writing – review & editing. IB: Conceptualization, Resources, Writing – review & editing. RW: Conceptualization, Formal analysis, Funding acquisition, Methodology, Resources, Software, Supervision, Validation, Writing – review & editing.
